# Supplementary material for: Heparin-based hydrogel scaffolding alters the transcriptomic profile and increases the chemoresistance of MDA-MB-231 triple-negative breast cancer cells
Source: Biomater Sci. 2020 Feb 13;8(10):2786–96. doi: 10.1039/c9bm01481k (PMC7497406; doi:10.1039/c9bm01481k)
Supplement: Supplementary file 2 [file BM-008-C9BM01481K-s002.zip › Supplementary File 4/HP-BhydrogelvControl/Pathways/my_analysis.Gsea.1545201079907/HALLMARK_APOPTOSIS.html]

Details for gene set HALLMARK\_APOPTOSIS[GSEA]

|  || Dataset | expr.class.cls#HYD\_versus\_CONTROL.class.cls#HYD\_versus\_CONTROL\_repos |
| Phenotype | class.cls#HYD\_versus\_CONTROL\_repos |
| Upregulated in class | CONTROL |
| GeneSet | HALLMARK\_APOPTOSIS |
| Enrichment Score (ES) | -0.34034836 |
| Normalized Enrichment Score (NES) | -1.5753969 |
| Nominal p-value | 0.0 |
| FDR q-value | 0.012245985 |
| FWER p-Value | 0.066 |
Table: GSEA Results Summary

  

Fig 1: Enrichment plot: HALLMARK\_APOPTOSIS      
 Profile of the Running ES Score & Positions of GeneSet Members on the Rank Ordered List

  

| PROBE | DESCRIPTION (from dataset) | GENE SYMBOL | GENE\_TITLE | RANK IN GENE LIST | RANK METRIC SCORE | RUNNING ES | CORE ENRICHMENT || 1 | CREBBP | na |  |  | 967 | 1.896 | -0.0313 | No |
| 2 | LEF1 | na |  |  | 1417 | 1.622 | -0.0382 | No |
| 3 | SMAD7 | na |  |  | 1487 | 1.581 | -0.0256 | No |
| 4 | RHOT2 | na |  |  | 1503 | 1.574 | -0.0102 | No |
| 5 | GADD45B | na |  |  | 1972 | 1.375 | -0.0207 | No |
| 6 | ERBB2 | na |  |  | 1977 | 1.374 | -0.0068 | No |
| 7 | JUN | na |  |  | 1992 | 1.366 | 0.0065 | No |
| 8 | DDIT3 | na |  |  | 2346 | 1.225 | 0.0006 | No |
| 9 | GADD45A | na |  |  | 2424 | 1.200 | 0.0089 | No |
| 10 | MADD | na |  |  | 2519 | 1.167 | 0.0159 | No |
| 11 | SPTAN1 | na |  |  | 2525 | 1.164 | 0.0276 | No |
| 12 | ATF3 | na |  |  | 2583 | 1.150 | 0.0364 | No |
| 13 | PPP2R5B | na |  |  | 3034 | 1.028 | 0.0233 | No |
| 14 | CDC25B | na |  |  | 3153 | 0.999 | 0.0274 | No |
| 15 | ERBB3 | na |  |  | 3335 | 0.957 | 0.0277 | No |
| 16 | FDXR | na |  |  | 3632 | 0.886 | 0.0213 | No |
| 17 | DIABLO | na |  |  | 3643 | 0.883 | 0.0298 | No |
| 18 | TNFRSF12A | na |  |  | 3645 | 0.883 | 0.0388 | No |
| 19 | PSEN2 | na |  |  | 3877 | 0.838 | 0.0353 | No |
| 20 | WEE1 | na |  |  | 4603 | 0.692 | 0.0044 | No |
| 21 | BMP2 | na |  |  | 4891 | 0.643 | -0.0041 | No |
| 22 | BAX | na |  |  | 4924 | 0.638 | 0.0008 | No |
| 23 | CFLAR | na |  |  | 4965 | 0.633 | 0.0052 | No |
| 24 | H1F0 | na |  |  | 5247 | 0.593 | -0.0035 | No |
| 25 | PLCB2 | na |  |  | 5576 | 0.541 | -0.0151 | No |
| 26 | IRF1 | na |  |  | 5648 | 0.533 | -0.0134 | No |
| 27 | EGR3 | na |  |  | 6013 | 0.483 | -0.0275 | No |
| 28 | CDKN1B | na |  |  | 6043 | 0.481 | -0.0241 | No |
| 29 | GCH1 | na |  |  | 6157 | 0.463 | -0.0253 | No |
| 30 | CASP9 | na |  |  | 6333 | 0.439 | -0.0300 | No |
| 31 | BMF | na |  |  | 6419 | 0.427 | -0.0300 | No |
| 32 | NEFH | na |  |  | 6997 | 0.360 | -0.0566 | No |
| 33 | SQSTM1 | na |  |  | 7012 | 0.359 | -0.0537 | No |
| 34 | IL18 | na |  |  | 7027 | 0.356 | -0.0507 | No |
| 35 | EBP | na |  |  | 7038 | 0.355 | -0.0476 | No |
| 36 | IL6 | na |  |  | 7244 | 0.333 | -0.0550 | No |
| 37 | TNF | na |  |  | 7258 | 0.331 | -0.0523 | No |
| 38 | CCND1 | na |  |  | 7494 | 0.308 | -0.0614 | No |
| 39 | TGFBR3 | na |  |  | 7615 | 0.290 | -0.0647 | No |
| 40 | HMGB2 | na |  |  | 7660 | 0.284 | -0.0641 | No |
| 41 | BTG2 | na |  |  | 7702 | 0.284 | -0.0634 | No |
| 42 | MCL1 | na |  |  | 7729 | 0.284 | -0.0618 | No |
| 43 | DFFA | na |  |  | 7761 | 0.279 | -0.0606 | No |
| 44 | RARA | na |  |  | 8244 | 0.227 | -0.0835 | No |
| 45 | BIK | na |  |  | 8297 | 0.220 | -0.0840 | No |
| 46 | CASP2 | na |  |  | 8779 | 0.169 | -0.1075 | No |
| 47 | ROCK1 | na |  |  | 9075 | 0.144 | -0.1215 | No |
| 48 | IL1B | na |  |  | 9136 | 0.136 | -0.1233 | No |
| 49 | RHOB | na |  |  | 9250 | 0.124 | -0.1279 | No |
| 50 | DAP3 | na |  |  | 9259 | 0.122 | -0.1271 | No |
| 51 | ANKH | na |  |  | 9443 | 0.098 | -0.1357 | No |
| 52 | CCND2 | na |  |  | 9609 | 0.088 | -0.1434 | No |
| 53 | IL1A | na |  |  | 9651 | 0.080 | -0.1448 | No |
| 54 | PDGFRB | na |  |  | 9922 | 0.051 | -0.1584 | No |
| 55 | IGF2R | na |  |  | 10025 | 0.038 | -0.1634 | No |
| 56 | PEA15 | na |  |  | 10142 | 0.023 | -0.1692 | No |
| 57 | TIMP2 | na |  |  | 10178 | 0.018 | -0.1709 | No |
| 58 | CASP1 | na |  |  | 10302 | 0.015 | -0.1772 | No |
| 59 | PTK2 | na |  |  | 10391 | 0.008 | -0.1817 | No |
| 60 | RETSAT | na |  |  | 10420 | 0.005 | -0.1831 | No |
| 61 | CCNA1 | na |  |  | 10576 | -0.014 | -0.1911 | No |
| 62 | DPYD | na |  |  | 10582 | -0.014 | -0.1912 | No |
| 63 | SLC20A1 | na |  |  | 10603 | -0.017 | -0.1921 | No |
| 64 | TAP1 | na |  |  | 10646 | -0.022 | -0.1941 | No |
| 65 | ADD1 | na |  |  | 10759 | -0.033 | -0.1996 | No |
| 66 | CTH | na |  |  | 10798 | -0.037 | -0.2012 | No |
| 67 | BCL2L2 | na |  |  | 10801 | -0.037 | -0.2010 | No |
| 68 | CASP8 | na |  |  | 10884 | -0.051 | -0.2048 | No |
| 69 | GNA15 | na |  |  | 10989 | -0.064 | -0.2096 | No |
| 70 | ENO2 | na |  |  | 11078 | -0.075 | -0.2134 | No |
| 71 | CD44 | na |  |  | 11081 | -0.075 | -0.2127 | No |
| 72 | CD14 | na |  |  | 11546 | -0.118 | -0.2359 | No |
| 73 | CDKN1A | na |  |  | 11746 | -0.145 | -0.2448 | No |
| 74 | NEDD9 | na |  |  | 12213 | -0.201 | -0.2672 | No |
| 75 | BRCA1 | na |  |  | 12249 | -0.206 | -0.2669 | No |
| 76 | FEZ1 | na |  |  | 12281 | -0.210 | -0.2664 | No |
| 77 | TSPO | na |  |  | 12292 | -0.212 | -0.2648 | No |
| 78 | FAS | na |  |  | 12828 | -0.261 | -0.2901 | No |
| 79 | BCAP31 | na |  |  | 12908 | -0.272 | -0.2915 | No |
| 80 | RELA | na |  |  | 13082 | -0.296 | -0.2975 | No |
| 81 | TIMP3 | na |  |  | 13429 | -0.342 | -0.3122 | No |
| 82 | SAT1 | na |  |  | 13558 | -0.362 | -0.3152 | No |
| 83 | SOD2 | na |  |  | 13651 | -0.378 | -0.3161 | No |
| 84 | ISG20 | na |  |  | 13723 | -0.391 | -0.3158 | No |
| 85 | APP | na |  |  | 13765 | -0.397 | -0.3139 | No |
| 86 | IGFBP6 | na |  |  | 13803 | -0.403 | -0.3117 | No |
| 87 | PLAT | na |  |  | 13867 | -0.414 | -0.3108 | No |
| 88 | CYLD | na |  |  | 14028 | -0.420 | -0.3148 | No |
| 89 | GPX4 | na |  |  | 14284 | -0.460 | -0.3235 | No |
| 90 | PSEN1 | na |  |  | 14410 | -0.478 | -0.3252 | No |
| 91 | BCL2L11 | na |  |  | 14467 | -0.486 | -0.3231 | No |
| 92 | TGFB2 | na |  |  | 14509 | -0.492 | -0.3202 | No |
| 93 | CLU | na |  |  | 14511 | -0.492 | -0.3152 | No |
| 94 | TIMP1 | na |  |  | 14560 | -0.499 | -0.3126 | No |
| 95 | SATB1 | na |  |  | 14770 | -0.532 | -0.3181 | No |
| 96 | GPX3 | na |  |  | 14790 | -0.536 | -0.3136 | No |
| 97 | BCL2L1 | na |  |  | 14898 | -0.549 | -0.3136 | No |
| 98 | GPX1 | na |  |  | 15409 | -0.635 | -0.3338 | Yes |
| 99 | HMOX1 | na |  |  | 15431 | -0.638 | -0.3284 | Yes |
| 100 | BID | na |  |  | 15537 | -0.659 | -0.3271 | Yes |
| 101 | GSR | na |  |  | 15538 | -0.659 | -0.3204 | Yes |
| 102 | GSN | na |  |  | 15576 | -0.665 | -0.3155 | Yes |
| 103 | ETF1 | na |  |  | 15634 | -0.675 | -0.3115 | Yes |
| 104 | HSPB1 | na |  |  | 15951 | -0.733 | -0.3206 | Yes |
| 105 | TOP2A | na |  |  | 15987 | -0.741 | -0.3148 | Yes |
| 106 | IFITM3 | na |  |  | 15996 | -0.744 | -0.3076 | Yes |
| 107 | KRT18 | na |  |  | 16203 | -0.788 | -0.3103 | Yes |
| 108 | CASP4 | na |  |  | 16359 | -0.824 | -0.3100 | Yes |
| 109 | CAV1 | na |  |  | 16368 | -0.825 | -0.3020 | Yes |
| 110 | RNASEL | na |  |  | 16540 | -0.865 | -0.3021 | Yes |
| 111 | TNFSF10 | na |  |  | 16587 | -0.869 | -0.2955 | Yes |
| 112 | PMAIP1 | na |  |  | 16591 | -0.869 | -0.2868 | Yes |
| 113 | XIAP | na |  |  | 16662 | -0.889 | -0.2813 | Yes |
| 114 | BCL10 | na |  |  | 16727 | -0.907 | -0.2754 | Yes |
| 115 | DAP | na |  |  | 16811 | -0.929 | -0.2702 | Yes |
| 116 | CTNNB1 | na |  |  | 16890 | -0.952 | -0.2645 | Yes |
| 117 | ANXA1 | na |  |  | 16924 | -0.963 | -0.2564 | Yes |
| 118 | BTG3 | na |  |  | 16989 | -0.983 | -0.2496 | Yes |
| 119 | EREG | na |  |  | 17124 | -1.024 | -0.2462 | Yes |
| 120 | CDK2 | na |  |  | 17319 | -1.090 | -0.2452 | Yes |
| 121 | DNM1L | na |  |  | 17464 | -1.139 | -0.2410 | Yes |
| 122 | BIRC3 | na |  |  | 17468 | -1.140 | -0.2295 | Yes |
| 123 | TXNIP | na |  |  | 17600 | -1.197 | -0.2241 | Yes |
| 124 | VDAC2 | na |  |  | 17787 | -1.281 | -0.2207 | Yes |
| 125 | PAK1 | na |  |  | 17820 | -1.295 | -0.2091 | Yes |
| 126 | PPP3R1 | na |  |  | 17995 | -1.378 | -0.2040 | Yes |
| 127 | CASP7 | na |  |  | 18210 | -1.506 | -0.1998 | Yes |
| 128 | F2R | na |  |  | 18280 | -1.550 | -0.1875 | Yes |
| 129 | IFNGR1 | na |  |  | 18491 | -1.701 | -0.1811 | Yes |
| 130 | CASP6 | na |  |  | 18493 | -1.701 | -0.1637 | Yes |
| 131 | PDCD4 | na |  |  | 18523 | -1.724 | -0.1475 | Yes |
| 132 | PPT1 | na |  |  | 18555 | -1.749 | -0.1312 | Yes |
| 133 | DNAJA1 | na |  |  | 18581 | -1.765 | -0.1144 | Yes |
| 134 | GSTM1 | na |  |  | 18616 | -1.803 | -0.0977 | Yes |
| 135 | CASP3 | na |  |  | 18683 | -1.879 | -0.0818 | Yes |
| 136 | BNIP3L | na |  |  | 18688 | -1.884 | -0.0627 | Yes |
| 137 | EMP1 | na |  |  | 18689 | -1.884 | -0.0434 | Yes |
| 138 | SOD1 | na |  |  | 18799 | -2.015 | -0.0284 | Yes |
| 139 | DNAJC3 | na |  |  | 18949 | -2.261 | -0.0130 | Yes |
| 140 | LGALS3 | na |  |  | 19056 | -2.536 | 0.0075 | Yes |
Table: GSEA details [plain text format]

  

Fig 2: HALLMARK\_APOPTOSIS      
 Blue-Pink O' Gram in the Space of the Analyzed GeneSet

  

Fig 3: HALLMARK\_APOPTOSIS: Random ES distribution      
 Gene set null distribution of ES for **HALLMARK\_APOPTOSIS**
